# Supplementary material for: A quantum encryption design featuring confusion, diffusion, and mode of operation
Source: Sci Rep. 2021 Dec 10;11:23774. doi: 10.1038/s41598-021-03241-8 (PMC8664820; doi:10.1038/s41598-021-03241-8)
Supplement: Supplementary file 1 — Supplementary Information. [file 41598_2021_3241_MOESM1_ESM.docx]

Supplementary information: A quantum encryption design featuring confusion, diffusion, and mode of operation

Zixuan Hu and Sabre Kais*

Department of Chemistry, Department of Physics, and Purdue Quantum Science and Engineering Institute, Purdue University, West Lafayette, IN 47907, United States
E-mail: [kais@purdue.edu](mailto:kais@purdue.edu)

**S1. Discussion on the avoidable scenario of Eve obtaining significant information on the ciphertext.**

In the main text we stated that even if the rare and avoidable scenario of Alice sending the same copy of the ciphertext many times does happen, and the adversary Eve can gain statistical knowledge of the ciphertext state, it will still be highly difficult for her to deduce the key and the plaintext. In the following we formalize this statement and present the details of the reasoning.

**Statement 1:** For any *n*-qubit ciphertext created by applying a polynomially long sequence *K* of 1-qubit and 2-qubit elementary gates on some *n*-qubit plaintext, suppose an adversary Eve can retrieve the coefficient associated with any basis state (e.g. she calls the basis state $|01010\rangle$ for a 5-qubit ciphertext, and get the coefficient $C_{01010}$), then she cannot deduce *K* within polynomial number of steps.

To understand Statement 1 we first cite the result from our previous study on quantum state complexity [1] that any sequence of 1-qubit and 2-qubit elementary gates is equivalent to a sequence of 2-qubit controlled-unitary gates or $C\left(U\right)$’s. All quantum states that can be created by polynomially long sequences of 1-qubit and 2-qubit elementary gates (or concisely all polynomial states) thus correspond to all the sequences of $C\left(U_{i}\right)$’s with the lengths smaller than $cn^{k}$ for some constant $c$ and $k$ such that $cn^{k}$ is overwhelmingly smaller than $2^{n}$. The complexity of a sequence of $C\left(U_{i}\right)$’s comes from the parameters used to define the $U_{i}$’s and the *configuration* of the sequence: each $C\left(U_{i}\right)$ has a control qubit and a target qubit that are selected from the *n* qubits, and the configuration of a sequence of length $cn^{k}$ is specified by the $cn^{k}$ number of control-target qubit pairs. Now suppose we are given a quantum state known to be created by a sequence of the length equal to $cn^{k}$, to determine the exact sequence used we need to first determine its configuration, and then the parameters of each $U_{i}$ can be determined by a system of equations defined by the configuration. The total possible number of configurations is $P\left(n,2\right)^{cn^{k}}=\left(n^{2}-n\right)^{cn^{k}}$ (where $P\left(n,2\right)$ is the permutation of choosing one control and one target out of *n* qubits), which is an extremely large number $\gg2^{n}$. If we consider the fact that the $C\left(U_{i}\right)$’s may commute, then the number of unique configurations will be smaller. It is hard to evaluate the effect of commutation without some knowledge of the $C\left(U_{i}\right)$’s in the sequence. However, in a particular example, we can design the sequence in a way that each $C\left(U_{i}\right)$ is non-commutative to the previous $C\left(U_{i-1}\right)$ and thus the number of unique configurations can be easily obtained. We assume the starting quantum state is created from $|0\rangle^{\otimes n}$ by applying a $U_{i}$ to each $q_{i}$ such that it is in a superposition between $|0\rangle$ and $|1\rangle$. We apply the sequence $\left(C\left(U_{1}\right)_{j\to k},C\left(U_{2}\right)_{k\to l},C\left(U_{3}\right)_{l\to h},C\left(U_{4}\right)_{h\to g},...\right)$ to the starting state, where the subscript $j\to k$ means $q_{j}$ is the control and $q_{k}$ is the target. In this sequence each $C\left(U_{i}\right)$ uses the target of the previous $C\left(U_{i-1}\right)$ as the control, which ensures that $C\left(U_{i}\right)$ is non-commutative to $C\left(U_{i-1}\right)$. As we can choose the initial $q_{j}$ from *n* qubits, the initial target $q_{k}$ from $n-1$ qubits (excluding $q_{j}$), and subsequent targets from $n-1$ qubits (excluding its own control), the total number of unique configurations is $n\left(n-1\right)^{cn^{k}}$. This example gives a lower bound on the number of unique configurations by restricting the control of each $C\left(U_{i}\right)$ to be the target of the previous $C\left(U_{i-1}\right)$. If we remove this restriction and consider commutation, then the actual possible number *M* of unique configurations falls in the range $n\left(n-1\right)^{cn^{k}}<M<\left(n^{2}-n\right)^{cn^{k}}$: clearly *M* is an extremely large number $\gg2^{n}$. Consequently it is impossible to determine the configuration within polynomial number of steps unless there is a very efficient way to sort through the extremely large number of possible configurations. Currently there is no efficient way to relate the coefficients of a quantum state to the configuration. Although we cannot decisively prove that there will never be a way to do so – indeed it is perhaps impossible to prove that something unspecified can never happen in the future – it is highly unlikely for the following reason. Firstly as the configuration contains the information on the control and target qubits of each $C\left(U_{i}\right)$, it also tells us how many $C\left(U_{i}\right)$ gates are used to create the state. Suppose such a method is developed such that Eve could determine the configuration of any polynomial quantum state within polynomial steps, then there must exist a collection of polynomial number of procedures (each of the procedures takes polynomial steps to perform) that she can perform on an arbitrary polynomial state and discover the configuration before the procedures are exhausted. This means that given a general *n*-qubit state, she can just perform these procedures on the state assuming as if it is polynomial, and if it gives a configuration before the procedures are exhausted, then we know how many steps are required to create the state; otherwise if it does not give a configuration before the procedures are exhausted, then it is not a polynomial state. Consequently the ability of Eve to efficiently determine the configuration of a polynomial state leads to her ability to tell if a general quantum state is polynomial or not. This is a contradiction to the result proved in our previous work [1] that it is exponentially hard to determine if a general quantum state can be created within polynomial number of gates. We therefore conclude that the key of our quantum encryption design is secure even if Eve has gained significant information on the ciphertext state. Furthermore, this compromising scenario of Alice sending the same copy of the ciphertext many times can be totally avoided by the confusion, diffusion, and mode of operation introduced in the main text.

**S2. A worked-out example of the encryption process illustrated in Figure 1 of the main text**

In Figure 1 and the associated discussions of the main text we illustrated an encrypting process that ensures confusion and diffusion. Here we present a worked-out example of a 4-qubit system capable of encrypting 4 bits of information.

**Preparation**: Initial state set to $|1010\rangle$ representing the plaintext 1010. The 16-dimensional state vector is $\left(0000000000100000\right)$.

**Step 1**: Apply a 1-qubit unitary $U_{i}$ to each qubit $q_{i}$ and create the initial dependence of each $q_{i}$ to its corresponding $U_{i}$. In this example we define

$\begin{matrix} U_{i}=\left(\begin{matrix} u_{i,1} & u_{i,2} \\ u_{i,2} & -u_{i,1} \end{matrix}\right),\text{ }u_{i,2}=\sqrt{1-u_{i,1}^{2}} \\ u_{1,1}=0.35\text{ }u_{2,1}=0.85\text{ }u_{3,1}=0.45\text{ }u_{4,1}=0.95 \end{matrix}$ (S)

When acting on the 16-dimensional state vector, the 16-by-16 matrix for e.g. $U_{1}$ is given by $M_{U_{1}}=U_{1}\otimes I\otimes I\otimes I$, where *I* is the 2-dimensional identity. The matrices for other $U_{i}$’s are calculated in a similar manner.

**Step 2**: Apply $\text{CNOT}_{1\to2}$, then $\text{CNOT}_{2\to3}$, then $\text{CNOT}_{3\to4}$. Like $U_{i}$ these CNOT gates are converted into 16-by-16 matrices before acting on the state vector (same for the following steps too)

**Step 3 and Step 4**: In the main text these two steps are discussed separately for easier understanding. In practice the two steps can be combined. Apply $\text{CNOT}_{4\to1}$, then $\text{CNOT}_{1\to3}$, then $\text{CNOT}_{3\to2}$.

**Results**: After Step 4 the ciphertext state vector is now $\left(\begin{matrix} 0.676,-0.0514,0.138,-0.252,-0.211,0.0418,-0.112,0.0788, \\ 0.0259,-0.340,0.127,-0.0693,-0.0830,0.419,-0.156,0.222 \end{matrix}\right)$. The probability of measuring $|0\rangle$ for each qubit is $p\left(q_{1}=|0\rangle\right)=0.607$, $p\left(q_{2}=|0\rangle\right)=0.679$, $p\left(q_{3}=|0\rangle\right)=0.804$, $p\left(q_{4}=|0\rangle\right)=0.580$.

**Confusion**: If we take $q_{2}$ for example, $p\left(q_{2}=|0\rangle\right)=0.130$ when $u_{2,1}$ only is changed to 0.2 (all other $u_{i,1}$ unchanged); and $p\left(q_{2}=|0\rangle\right)=0.295$ when $u_{4,1}$ only is changed to 0.2 (all other $u_{i,1}$ unchanged). So $q_{2}$ depends on more than half of the $U_{i}$’s that are parts of the key. All other qubits have similar results and confusion is achieved.

**Diffusion**: For example, if we change the plaintext by one bit to 1011, the ciphertext state vector becomes $\left(\begin{matrix} 0.222,0.156,-0.419,-0.0830,-0.0693,-0.127,0.340,0.0259, \\ -0.0788,-0.112,0.0418,0.211,0.252,0.138,-0.0514,-0.676 \end{matrix}\right)$. The probability of measuring $|0\rangle$ for each qubit changes to $p\left(q_{1}=|0\rangle\right)=0.393$, $p\left(q_{2}=|0\rangle\right)=0.321$, $p\left(q_{3}=|0\rangle\right)=0.196$, $p\left(q_{4}=|0\rangle\right)=0.420$. Compared with the previous results of $p\left(q_{1}=|0\rangle\right)=0.607$, $p\left(q_{2}=|0\rangle\right)=0.679$, $p\left(q_{3}=|0\rangle\right)=0.804$, $p\left(q_{4}=|0\rangle\right)=0.580$, we see that changing just one bit in the plaintext causes the probabilities of measuring more than half of the qubits to change. Changing other bits of the plaintext produce similar results and diffusion is achieved.

**S3. A worked-out example of the quantum mode of operation illustrated in Figure 2 of the main text**

In Figure 2 and the associated discussions of the main text we illustrated a quantum mode of operation. Here we present a worked-out example based on the same 4-qubit system and encryption procedure as detailed in the previous Section S2. Assume the plaintext of the 1^st^ block is 1001, and the initialization vector (IV) is 0011, then the altered plaintext is:

$P_{1}\oplus IV=1001\oplus0011=1010$ (S)

Now apply the same encryption procedure as in Section S2, after Step 4 the 1^st^ ciphertext state vector is:

$|C_{1}\rangle=E_{K}\left(P_{1}\oplus\text{IV}\right)=\left(\begin{matrix} 0.676,-0.0514,0.138,-0.252,-0.211,0.0418,-0.112,0.0788, \\ 0.0259,-0.340,0.127,-0.0693,-0.0830,0.419,-0.156,0.222 \end{matrix}\right)$ (S)

Now we create another copy of $|C_{1}\rangle$ and measure it to produce $M\left(|C_{1}\rangle\right)$ which is a random bit string. Without loss of generality, we may arbitrarily assume $M\left(|C_{1}\rangle\right)=0110$. Now suppose the plaintext of the 2^nd^ block is 1110, then the altered plaintext is:

$P_{2}\oplus M\left(|C_{1}\rangle\right)=1110\oplus0110=1000$ (S)

Now apply the same encryption procedure as in Section S2, after Step 4 the 2^nd^ ciphertext state vector is:

$|C_{2}\rangle=E_{K}\left(P_{2}\oplus M\left(|C_{1}\rangle\right)\right)=\left(\begin{matrix} 0.340,-0.0259,0.0693,-0.127,0.419,-0.0830,0.222,-0.156 \\ -0.0514,0.676,-0.252,0.138,-0.0418,0.211,-0.0788,0.112 \end{matrix}\right)$ (S)

Again we create another copy of $|C_{2}\rangle$ and measure it to produce $M\left(|C_{2}\rangle\right)$ which is a random bit string. Without loss of generality, we may arbitrarily assume $M\left(|C_{2}\rangle\right)=1100$. Suppose the plaintext of the 3^rd^ block is 0011, then the altered plaintext is:

$P_{3}\oplus M\left(|C_{2}\rangle\right)=0011\oplus1100=1111$ (S)

With the same encryption procedure, after Step 4 the 3^rd^ ciphertext state vector is:

$|C_{3}\rangle=E_{K}\left(P_{3}\oplus M\left(|C_{2}\rangle\right)\right)=\left(\begin{matrix} 0.138,-0.252,0.676,-0.0514,0.112,-0.0788,0.211,-0.0418, \\ 0.127,-0.0693,0.0259,-0.340,0.156,-0.222,0.0830,-0.419 \end{matrix}\right)$ (S)

Now suppose we stop at the 3^rd^ block, and send IV, $|C_{1}\rangle$, $M\left(|C_{1}\rangle\right)$, $|C_{2}\rangle$, $M\left(|C_{2}\rangle\right)$, and $|C_{3}\rangle$ to the recipient Bob. Bob first decrypts with $E_{K}^{-1}\left(|C_{1}\rangle\right)=P_{1}\oplus\text{IV=1010}$, and then get $P_{1}=P_{1}\oplus\text{IV}\oplus\text{IV=1001}$. He then decrypts with $E_{K}^{-1}\left(|C_{2}\rangle\right)=P_{2}\oplus M\left(|C_{1}\rangle\right)=1000$, and get $P_{2}=P_{2}\oplus M\left(|C_{1}\rangle\right)\oplus M\left(|C_{1}\rangle\right)=1110$. Finally he decrypts with $E_{K}^{-1}\left(|C_{3}\rangle\right)=P_{3}\oplus M\left(|C_{2}\rangle\right)=1111$, and then get $P_{3}=P_{3}\oplus M\left(|C_{2}\rangle\right)\oplus M\left(|C_{2}\rangle\right)=0011$. We see that Bob has successfully recovered all the three plaintexts.

References:

1. Hu, Z. and S. Kais, *Characterization of Quantum States Based on Creation Complexity.* Advanced Quantum Technologies, 2020. **n/a**(n/a): p. 2000043.
